# The meaning of screening: detection of brain metastasis in the adjuvant setting for stage III melanoma

**DOI:** 10.1016/j.esmoop.2022.100600

**Published:** 2022-10-17

**Authors:** S.H.A.E. Derks, K. de Joode, E.E.A.P. Mulder, L.S. Ho, A. Joosse, M.J.A. de Jonge, C. Verhoef, D.J. Grünhagen, M. Smits, M.J. van den Bent, A.A.M. van der Veldt

**Affiliations:** 1 Department of Neuro-Oncology, Erasmus MC Cancer Institute, Rotterdam, the Netherlands; 2 Department of Medical Oncology, Erasmus MC Cancer Institute, Rotterdam, the Netherlands; 3 Department of Radiology & Nuclear Medicine, Erasmus MC, Rotterdam, the Netherlands; 4 Department of Surgical Oncology, Erasmus MC Cancer Institute, Rotterdam, the Netherlands

**Keywords:** brain metastasis, melanoma, stage III, adjuvant treatment, screening, MRI of the brain, nivolumab, pembrolizumab, dabrafenib–trametinib

## Abstract

**Background:**

The incidence of melanoma is increasing and 37% of patients with metastatic melanoma eventually have brain metastasis (BM). Currently, there is no consensus on screening for BM in patients with resected stage III melanoma. However, given the high incidence of BM, routine screening magnetic resonance imaging (MRI) of the brain is considered in patients with completely resected stage III melanoma before the start of adjuvant treatment. The aim of this study was to assess the yield of screening for BM in these patients.

**Materials and methods:**

A single-center cohort study was carried out in the Erasmus MC, Rotterdam, The Netherlands, a large tertiary referral center for patients with melanoma. Eligible patients with complete resection of stage III melanoma and a screening MRI of the brain, made within 12 weeks after resection and before adjuvant treatment (programmed cell death protein 1 inhibitors, dabrafenib–trametinib), available between 1 August 2018 and 1 January 2021, were included.

**Results:**

A total of 202 patients were included. Eighteen (8.9%) of 202 patients had extracranial metastasis at screening. Two (1.1%) of the remaining 184 patients had BM at screening, resulting in a switch from adjuvant treatment to ipilimumab–nivolumab. At a median follow-up of 21.2 months, BM was detected in another 4 (2.4%) of 166 patients who started with adjuvant treatment.

**Conclusions:**

The yield of screening MRI of the brain is low after complete resection of stage III melanoma, before the start of adjuvant treatment. Therefore, routine screening MRI is not recommended in this setting.

## Highlights

• The yield of screening MRI of the brain is low in resected stage III melanoma.
• The incidence of extracranial metastasis is relatively high in this setting.
• Before adjuvant treatment, screening for extracranial metastasis is recommended.
• Routine screening MRI for the detection of brain metastasis is not recommended.

## Introduction

Cutaneous melanoma is listed in the top 20 of most common cancers worldwide, with 324 635 new cases and 57 043 deaths globally reported in 2020.^1^ The incidence of melanoma is increasing, with 3%-7% more cases per year.^2^^,^^3^ In ∼25% of all patients with melanoma, metastatic disease (stage IV) is eventually diagnosed and in 37% of patients with metastatic disease, brain metastasis (BM) is detected.^4^^,^^5^

In 2018, the European Medicines Agency (EMA) approved adjuvant treatments with monotherapy anti-programmed cell death protein 1 (PD-1) (nivolumab and pembrolizumab) and targeted therapy (dabrafenib–trametinib) for patients after complete resection of stage III melanoma.^6^, ^7^, ^8^ However, ∼18% of patients with stage III melanoma have disease recurrence within 12 weeks after complete resection, before starting with adjuvant treatment.^9^ Since patients with stage III and IV melanoma are treated with different therapeutic strategies, adequate disease staging is essential to optimize the treatment regimen for individual patients. Therefore, in patients with resected stage III melanoma, restaging before the start of adjuvant treatment is recommended and was also carried out in the pivotal trials.^6^, ^7^, ^8^ Although consensus exists for the need to screen for extracranial metastasis (ECM) after complete resection of stage III melanoma, screening for BM is not routinely carried out.

While international guidelines recommend screening of BM in stage IV melanoma, there is no consensus for screening of BM in stage III melanoma.^10^, ^11^, ^12^, ^13^ The National Comprehensive Cancer Network (NCCN) and the European Society for Medical Oncology (ESMO) state that screening with magnetic resonance imaging (MRI) for patients with stage IIB and higher is optional, whereas the recent 2022 European consensus-based interdisciplinary guideline recommends screening in all patients with stage III and IV.^10^^,^^11^^,^^13^ The National Institute for Health and Care Excellence (NICE) recommends screening in stage IV, but not specifically in lower stages.^12^ Remarkably, the timing of screening is not clearly determined and differs between guidelines. The ESMO guideline recommends screening for BM before complete resection of stage III, whereas the NCCN guideline recommends screening every 3-6 months after resection.^10^^,^^11^ Furthermore, the pivotal trials on adjuvant treatment in resected stage III melanoma used different inclusion criteria: in the COMBI-AD (dabrafenib–trametinib) and CheckMate-238 (nivolumab) trials, MRI of the brain was required before inclusion and start of adjuvant treatment,^7^^,^^8^ whereas this was not routinely carried out in the KeyNote-054 trial (pembrolizumab).^6^

Patients with stage III melanoma have a considerable risk of developing BM, with a lifetime risk between 18.5% and 23.5%.^5^^,^^14^ Therefore, following a consensus of melanoma experts in the Netherlands, MRI of the brain was implemented as standard of care in patients with completely resected stage III melanoma before the start of adjuvant treatment. However, routine use of MRI substantially impacts health care capacity and costs, while it remains unclear whether screening for BM has additional value for patients in this particular setting. Therefore, we assessed the yield of screening MRI of the brain before the start of adjuvant treatment in patients with resected stage III melanoma and evaluated the clinical impact of detecting BM for individual patients.

## Materials and methods

### Patient selection

This retrospective study was carried out at the Erasmus MC, Rotterdam, The Netherlands, which is a large tertiary referral center for melanoma. Based on the reimbursement in the Netherlands of adjuvant treatment in 2018, we identified all consecutive patients with cutaneous and mucosal melanoma with complete resection of stage III melanoma who were restaged between 1 August 2018 and 1 January 2021, allowing a minimum follow-up duration of 12 months. Only patients who were referred for adjuvant treatment after complete resection of stage III melanoma and who underwent screening MRI of the brain before adjuvant treatment were included in the present analysis. The study was approved by the local institutional review board (MEC-2021-0735).

### Restaging and surveillance protocol

According to the standard of care at the Erasmus MC, all selected patients underwent complete resection and subsequent restaging within 12 weeks before the start of adjuvant treatment. For this restaging, computed tomography (CT) or [^18^F]2-fluoro-2-deoxy-d-glucose (FDG)–positron emission tomography (PET)–CT was carried out for screening of ECM, whereas MRI of the brain was carried out for screening of BM. Screening MRI routinely included transversal T1-weighted imaging pre- and post-gadolinium contrast administration (with an effective slice thickness of 3 mm and 0.8 mm, respectively), T2-weighted imaging, and diffusion-weighted imaging. Acquired images were assessed by experienced neuroradiologists. After the start of adjuvant treatment, follow-up imaging consisted of CT (thorax/abdomen) every 3-4 months in the first 2 years and at least one ^18^F-FDG–PET at 2 years.^15^^,^^16^ During follow-up, MRI of the brain was only repeated to monitor lesions which were newly detected on screening MRI, at recurrence of ECM, or in case of symptoms of BM such as headache, epilepsy, or neurologic deficits.

### Data collection

Patient-related characteristics and treatments were collected from the electronic patient files. For all patients, disease stage was determined according to the eighth edition of the American Joint Committee on Cancer.^17^ In line with the inclusion criteria of the pivotal trials, at least one micrometastasis >1 mm in largest diameter in the sentinel node for N1a was required for stage IIIA.^6^^,^^7^

All imaging results were collected from clinical radiology reports. For quality control, an experienced neuroradiologist (MS) who was blinded to clinical outcome carried out a second read in a sample of 34 patients with a negative first read and a high risk of BM, i.e. patients with stage IIIC disease. In none of these 34 patients, BM was detected during this second read.

### Outcomes and statistical analysis

All intracranial lesions on MRI were either histopathologically examined or monitored with follow-up MRI to determine whether these lesions were true or false positive for BM. The primary outcome was the number of patients with BM detected by screening MRI at restaging before the start of adjuvant treatment. Secondary outcomes were the clinical impact of BM detected by screening MRI at restaging (i.e. change of treatment plan), the number of patients with ECM detected by screening CT or PET at restaging, and the number of patients with BM detected by MRI carried out on indication during follow-up.

## Results

### Patient selection

Between August 2018 and January 2021, 368 consecutive patients with any stage melanoma underwent screening MRI of the brain (Supplementary Figure S1, available at https://doi.org/10.1016/j.esmoop.2022.100600). Of these 368 patients, 166 patients were excluded because of other disease stages (*n* = 161) or because they had received neoadjuvant treatment (*n* = 5). In total, 202 patients with stage III melanoma and screening MRI of the brain within 12 weeks after complete resection were selected for the current analysis.

### Baseline characteristics

**Figure 1:**
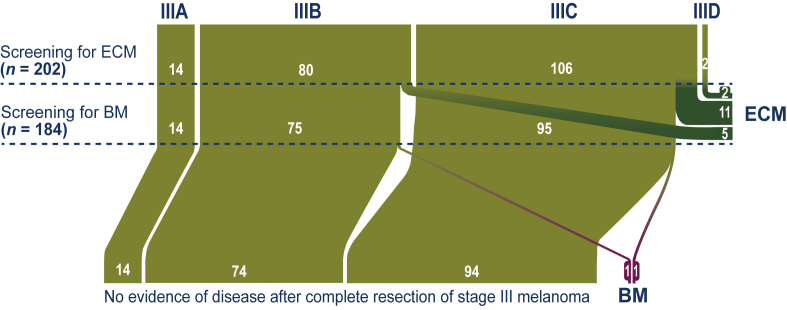
**Bar chart of all patients with complete resection of stage III melanoma at restaging before adjuvant treatment (*n* = 202).** Eighteen (8.9%) of 202 patients had extracranial metastasis (ECM) at restaging. Two (1.1%) of the remaining 184 patients had solitary brain metastasis (BM) at restaging.

Of the 202 patients, median age at screening MRI of the brain (baseline) was 63.5 years (Q1 56.5-Q3 72.5 years) and 112 (55.4%) of 202 patients were male (Table 1). Fourteen (6.9%) of 202 patients were diagnosed with stage IIIA, 80 (39.6%) patients with stage IIIB, 106 (52.5%) patients with stage IIIC, and 2 (1.0%) patients with stage IIID melanoma (Table 1, Figure 1). *BRAF* mutation status was determined in 138 (68.3%) of 202 patients and a *BRAF* V600E/K mutation was detected in 80 (58.0%) of these 138 patients. The median study follow-up, between screening MRI of the brain and time of analysis, was 21.7 months (Q1 15.9-Q3 29.0 months).

**Table 1:** Baseline characteristics of patients (*n* = 202) with completely resected stage III melanoma and screening MRI of the brain before adjuvant treatment

Variable	No. of patients	Proportion (%)
	202	100
Median age (years, Q1-Q3)	63.5	(56.5-72.5)
Male	112	55.4
Disease stage		
Stage IIIA	14	6.9
Stage IIIB	80	39.6
Stage IIIC	106	52.5
Stage IIID	2	1.0
*BRAF* status unknown	64	31.7
*BRAF* status determined	138	68.3
V600E/K	80	39.6^a^
Wild type	51	25.2^a^
Mutation of unknown significance	7	3.5^a^

MRI, magnetic resonance imaging.

a *BRAF* status is displayed as the proportion of all patients (*n* = 138) who had been tested for this mutation.

### Screening MRI before adjuvant treatment

Screening for ECM and BM was carried out as a part of routine restaging within the 12 weeks after complete resection and before the start of adjuvant treatment for patients with completely resected stage III melanoma (Figure 1). This resulted in the detection of ECM in 18 (8.9%) of 202 patients and 2 of these patients with ECM had concurrent BM. In the remaining 184 patients without ECM, lesions suspected for BM were detected in 9 (4.9%) patients. In seven (78%) of these nine patients, the lesions were false positive. One of these patients had a histopathologically confirmed glioblastoma as a second malignancy. The other six patients ultimately had benign lesions (e.g. vascular structure, meningioma) on follow-up MRI. Therefore, in 2 (1.1%) of 184 patients without ECM at restaging, screening MRI of the brain detected true, solitary BM (Supplementary Figure S2, available at https://doi.org/10.1016/j.esmoop.2022.100600). One of these two patients with solitary BM was originally staged with IIIB and the other with stage IIIC disease (Supplementary Table S1, available at https://doi.org/10.1016/j.esmoop.2022.100600).

For these two patients with BM at restaging, the treatment plan was changed from adjuvant treatment to combination therapy with nivolumab–ipilimumab. Local treatment of BM was not indicated since the BMs were small and asymptomatic.

### MRI during follow-up

Overall, 166 (91.2%) of the 182 patients without ECM or BM at restaging started with adjuvant treatment (Table 2). Adjuvant treatment consisted of anti-PD-1 monotherapy in 151 (91.0%) and dabrafenib–trametinib in 15 (9.0%) of 166 patients. During adjuvant treatment and subsequent follow-up, 51 (30.7%) of 166 patients had one or more MRI of the brain (Table 3). Of these 51 patients, 32 (62.7%) patients underwent MRI of the brain because of new ECM, 7 (13.7%) patients for symptoms of BM, 4 (7.8%) patients for a combination of ECM and symptoms, and 8 (15.7%) patients for other reasons (Table 3). In 4 (2.4%) of 166 patients, MRI detected BM at a median follow-up of 21.2 months (range 6.9-33.6 months) since initial screening MRI (Supplementary Table S1, available at https://doi.org/10.1016/j.esmoop.2022.100600). Two of these four patients with BM during follow-up had concurrent ECM, one patient had symptomatic BM, and one had both. One of these four patients with BM was originally diagnosed with stage IIIB and the other three patients with stage IIIC melanoma. All four patients with BM during follow-up had been treated with adjuvant anti-PD-1 and at diagnosis of BM, treatment was switched to dabrafenib–trametinib (*n* = 2), encorafenib–binimetinib (*n* = 1), and ipilimumab–nivolumab combined with neurosurgical resection (*n* = 1).

**Table 2:** Adjuvant treatment initiated after screening for BM in patients with complete resection of stage III melanoma (*n* = 166)

Variable	No. of patients	Proportion (%)
Adjuvant treatment	166	100
Anti-PD-1	151	91.0
Nivolumab	130	78.3
Pembrolizumab	21	12.7
Dabrafenib–trametinib	15	9.0

BM, brain metastasis; PD-1, programmed cell death protein 1.

**Table 3:** Patients with MRI of the brain during follow-up of adjuvant treatment (*n* = 51)

Variable	No. of patients	Proportion (%)
Patients with MRI of the brain during follow-up	51	100
Indications		
ECM	32	62.7
Symptoms suspected for BMs	7	13.7
Symptoms and ECM	4	7.8
Other	8	15.7

In eight patients, MRI had been carried out for other reasons (e.g. symptoms suspected of hypophysitis, follow-up of benign lesions).

BM, brain metastasis; ECM, extracranial metastasis; MRI, magnetic resonance imaging.

In the remaining 16 (8.8%) of 182 patients without ECM or BM at restaging, originally diagnosed with stage IIIA (*n* = 3), IIIB (*n* = 8), and IIIC (*n* = 5) melanoma, adjuvant treatment was not initiated. Eight patients had a contraindication for adjuvant treatment because of comorbidities (e.g. autoimmune disease, kidney transplant) and the other eight patients, two of whom with spontaneous remission after initial biopsy and histopathological diagnosis of stage III melanoma, preferred to be followed up. Of all 16 patients, 2 (12.5%) patients ultimately had BM with concurrent ECM during follow-up.

### Characteristics of brain metastasis

In the total of eight patients with BM at restaging within the 12 weeks after complete resection and before the intended start of adjuvant treatment (*n* = 2), during adjuvant treatment (*n* = 4), and during follow-up without adjuvant treatment (*n* = 2), the BMs had a median maximum diameter of 3.3 mm (range 2.2-36.3 mm) (Supplementary Table S1, available at https://doi.org/10.1016/j.esmoop.2022.100600). All patients had a solitary BM, except for one patient with 20 BMs.

## Discussion

The current study shows that at restaging within 12 weeks after complete resection of stage III melanoma and before the start of adjuvant treatment, ECM was detected in 8.9% of patients, whereas only small, solitary BMs were detected in 1.1% of patients without ECM.

The detection of BM changed the adjuvant treatment plan to nivolumab–ipilimumab. Early detection of melanoma BM may improve outcome since patients with asymptomatic BM and a better performance status have a higher chance of intracranial response to immune checkpoint inhibition.^18^^,^^19^ In addition, local therapies such as surgical resection or stereotactic radiotherapy have shown better intracranial control of smaller BMs, further indicating the need for early detection.^20^^,^^21^ However, in the current study, screening MRI detected very small (<5.5 mm), asymptomatic, and solitary BMs in only two patients. Both patients were not treated with local therapy, but with nivolumab–ipilimumab. This combination has a higher intracranial response rate compared to anti-PD-1 monotherapy.^19^^,^^22^

After the start of adjuvant treatment, the proportion of patients in whom BM were found remained low (2.4%), with BM detected at a median follow-up of 21.2 months. In a prospective study, in which patients with stage IIB-C and III melanoma had routine MRI of the brain every 6 months, BM was most frequently detected (1.1%) within the first 2 years after resection.^23^ In the current study, MRI of the brain was only carried out when indicated; therefore, the true incidence of (asymptomatic) BM during follow-up could be higher. Another retrospective study, without a well-defined imaging follow-up protocol, reported a much higher incidence (22.9%) of BM in stage III melanoma at a median follow-up of 20 months after resection.^14^ This higher incidence could be explained by the period of inclusion (2011-2017), which was before the introduction of adjuvant treatment in the clinic, and the longer study follow-up period with less optimal disease control.

In contrast to the low BM incidence, a relatively large proportion (8.9%) of patients had ECM at restaging before adjuvant treatment. In this same setting, Bloemendal et al. reported an even higher incidence (18%) for recurrent melanoma, while routine screening for BM was not carried out in their prospective study.^9^ This higher incidence could be partly explained by the exclusion of patients with stage IIIA. Our study confirms that the incidence of ECM is high within 12 weeks of complete resection and that screening for ECM is therefore recommended for restaging before adjuvant treatment.

Of all 182 patients without ECM or BM at restaging, 16 (8.8%) patients did not start with adjuvant treatment. These patients had a (relative) contraindication for adjuvant treatment or preferred conservative follow-up. Although it could have been considered to omit restaging in these patients, the detection of recurrent disease would have had a significant impact, thereby potentially changing the considerations for treatment.

Notably, in the vast majority of patients, no BMs were detected at screening or during follow-up in this study. The extensive use of MRI poses a substantial burden on health care costs and capacity. However, MRI is the method of choice as stated in the ESMO guideline on brain metastases from solid tumors.^24^ In seven patients, additional follow-up MRI or histopathological analysis determined false-positive findings on screening MRI of the brain. Furthermore, fear of imaging and scanxiety are well-known problems of cancer screening programs, as is the impact of incidental findings on brain MRI.^25^^,^^26^ Given the low yield of screening MRI for BM, these side-effects are disproportional to the potential benefit and should be prevented. Although we did not study cost-effectiveness, the low incidence of BM indicates that routine MRI of the brain is not justified at restaging before adjuvant treatment.

The retrospective design of this study is a limitation. Nevertheless, we carefully identified all screened patients. As a result, this study reflects daily clinical practice of a tertiary referral center for melanoma, where local guidelines for screening MRI of the brain were routinely applied to consecutive patients with resected stage III melanoma. Therefore, in the absence of prospective data, the findings of this study establish the limited usefulness of screening MRI of the brain in this clinical setting.

### Conclusions

The incidence of ECM is relatively high after complete resection of stage III melanoma. Consequently, restaging with (PET–) CT is recommended to detect ECM before adjuvant treatment. Since patients with ECM are known to have a high risk of BM, screening MRI of the brain is routinely carried out when ECM is detected. However, the yield of screening MRI of the brain is low in patients with completely resected stage III melanoma. Therefore, routine screening MRI to detect BM is not recommended before the start of adjuvant treatment.
