# Supplementary figures and images for: The meaning of screening: detection of brain metastasis in the adjuvant setting for stage III melanoma
Source: ESMO Open. 2022 Oct 17;7(6):100600. doi: 10.1016/j.esmoop.2022.100600 (PMC9808474; doi:10.1016/j.esmoop.2022.100600)

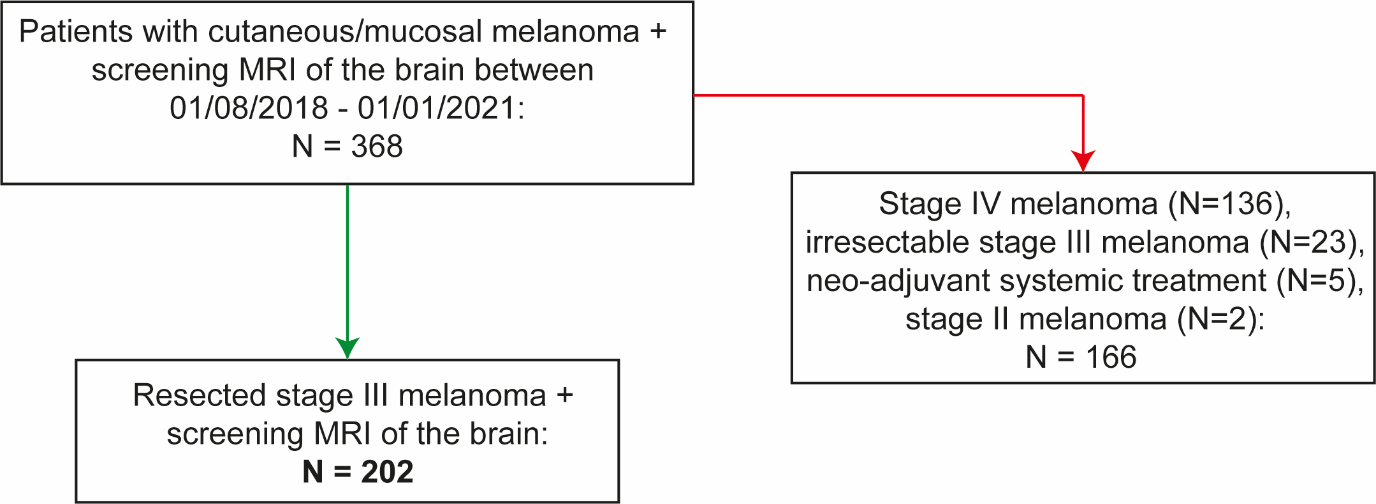


**Figure S1**. Flow chart of patient inclusion.

Supplement: Supplementary Figure S1 [file mmc1.docx]
