# Supplementary material for: The meaning of screening: detection of brain metastasis in the adjuvant setting for stage III melanoma
Source: ESMO Open. 2022 Oct 17;7(6):100600. doi: 10.1016/j.esmoop.2022.100600 (PMC9808474; doi:10.1016/j.esmoop.2022.100600)
Supplement: Supplementary Figure S2 [file mmc2.docx]

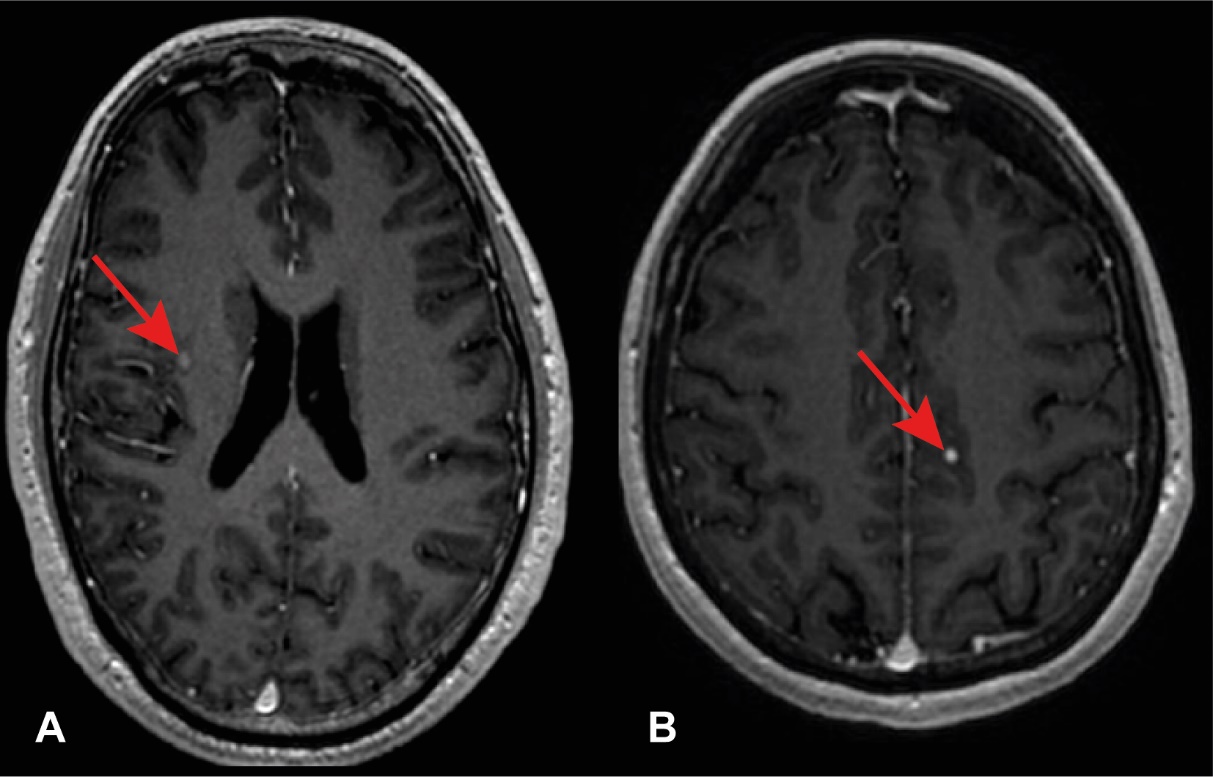


**Figure S2**. T1-weighted post-contrast MRI of the two patients (A, B) with a solitary brain metastasis (BM) and without extracranial metastasis (ECM) at restaging prior to adjuvant treatment.
