# Supplementary material for: The meaning of screening: detection of brain metastasis in the adjuvant setting for stage III melanoma
Source: ESMO Open. 2022 Oct 17;7(6):100600. doi: 10.1016/j.esmoop.2022.100600 (PMC9808474; doi:10.1016/j.esmoop.2022.100600)
Supplement: Supplementary Table S1 [file mmc3.docx]

| **Patient** | **Stage** | **No. of BMs** | **Largest diameter (mm)** | **Time (months) between screening MRI and BM** |
| --- | --- | --- | --- | --- |
| *BMs in 184 patients at screening* | | | |  |
| 1 | IIIB | 1 | 3.5 | 0 |
| 2 | IIIC | 1 | 5.3 | 0 |
| *BMs in 166 patients during follow-up of adjuvant treatment* | | | |  |
| 1 | IIIB | 1 | 7.8* | 33.6 |
| 2 | IIIC | 1 | 36.3 | 11.5 |
| 3 | IIIC | 1 | 2.2 | 6.9 |
| 4 | IIIC | 20 | 3.0 | 30.8 |
| *BMs in 16 patients during follow-up without adjuvant treatment* | | | | |
| 1 | IIIB | 1 | 3.0 | 13.6 |
| 2 | IIIB | 1 | 3.0 | 17.3 |

**Table S1**. Characteristics of brain metastasis (BM) of patients with completely resected stage III melanoma without extracranial metastasis (ECM) at restaging (N=184). The table was split according to time of diagnosis of BM: at screening within 12 weeks after complete resection of stage III melanoma, during follow-up of adjuvant treatment and during follow-up without adjuvant treatment. *This patient was also diagnosed with leptomeningeal disease.
